# Differences in Gut Microbes Across Age and Sex Linked to Metabolism and Microbial Stability in a Hibernating Mammal

**DOI:** 10.1002/ece3.70519

**Published:** 2024-11-10

**Authors:** Madison Pfau, Samuel Degregori, Paul H. Barber, Daniel T. Blumstein, Conner S. Philson

**Affiliations:** ^1^ Department of Ecology and Evolutionary Biology UCLA Los Angeles California USA; ^2^ Department of Environmental Science, Policy, and Management University of California, Berkeley Berkeley California USA; ^3^ Department of Anthropology Northwestern University Evanston Illinois USA; ^4^ Rocky Mountain Biological Laboratory Crested Butte Colorado USA; ^5^ Centre for Research in Animal Behaviour University of Exeter Exeter UK

**Keywords:** gut microbiome, maternal investment, sex differences, somatic investment

## Abstract

The gut microbiome has a well‐documented relationship with host fitness, physiology, and behavior. However, most of what is known comes from captive animals where diets and environments are more homogeneous or controlled. Studies in wild populations that experience dynamic environments and have natural life history variation are less common but are key to understanding the drivers of variation in the gut microbiome. Here we examine a wild population of yellow‐bellied marmots (*Marmota flaviventer*), an obligate winter hibernator, to quantify multivariate associations between host‐associated factors (e.g., age, sex, environmental harshness, and social behavior) and gut microbial composition. Across 5 years and 143 individuals, we found that males had a higher relative abundance of microbes associated with mass gain and cellulose digestion, which suggests a metabolic investment in mass gain (such as phylum *Firmicutes* and family *Lachnospiraceae*). By contrast, females had higher relative abundances of microbes associated with inflammation and metabolism (from microbial groups such as *Tenericutes* and *Ruminococcus*), possibly reflecting the importance of lactation and offspring investment. Post hoc analyses of lactating females showed a negative relationship with the abundance of microbes associated with mass gain but a positive relationship with microbes associated with metabolic energy, suggesting a trade‐off between investment in pups and maternal mass gain. Older animals also had reduced *Proteobacteria* relative abundance, a phylum associated with reduced inflammation. Results demonstrate that sex and age‐based traits, not sociality or environmental harshness, are associated with microbe‐mediated metabolism and inflammation in a wild, hibernating mammal.

## Graphical abstract

The gut microbiome has a well‐documented relationship with host fitness, physiology, and behavior. Here we examine a wild population of yellow‐bellied marmots (*Marmota flaviventer*), an obligate winter hibernator, to quantify multivariate associations between host‐associated factors (e.g., age, sex, environmental harshness, and social behavior), and gut microbial composition. Results demonstrate that sex and age‐based traits, not sociality or environmental harshness, are associated with microbe‐mediated metabolism and inflammation in a wild, hibernating mammal.

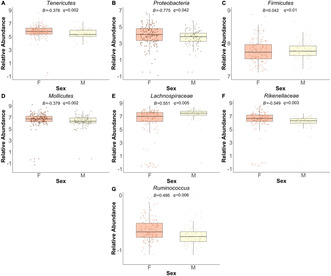

## 1 Introduction

Symbiotic gut microorganisms influence a suite of host fitness correlates, including metabolism, development, nutrient processing, behavior, reproductive success, and lifespan (Turnbaugh et al. 2006; Neish 2009; Cryan et al. 2019). However, the majority of gut microbiome studies use captive animals with controlled diets and homogenous environmental exposure (Clayton et al. 2016; Rosshart et al. 2019). Captive individuals have been shown to differ from their wild counterparts in microbial compositions (Bowerman et al. 2021; Reese et al. 2021). Indeed, captive individuals which are subsequently reintroduced into the wild have large shifts in microbial diversity and composition (Feng et al. 2022), highlighting the inability of captive animals to reflect the complexity of drivers of gut microbiome diversity. Studying factors associated with gut microbial composition in the wild will complement the work done in laboratory systems to create a more comprehensive understanding of the link between fitness and the gut microbiome.

In response to our growing understanding of the microbiome's relevance to individual fitness, it is important to study these associations in wild animals (Trevelline et al. 2019; Comizzoli et al. 2021). However, determining factors that influence microbial composition has proven difficult because there are both intrinsic and extrinsic factors that structure microbial communities (Falony et al. 2016; Miller, Svanbäck, and Bohannan 2018). Therefore, large‐scale studies of microbial metacommunities are needed to pinpoint possible factors associated with gut microbiome variation in wild populations (Miller, Svanbäck, and Bohannan 2018).

Previous studies show that gut microbial composition is shaped by an interplay between host genetics, maternal and social transmission, and environmental uptake (Rawls et al. 2006; Muegge et al. 2011; Grieneisen et al. 2019; Renelies‐Hamilton et al. 2021). Environmental factors, such as temperature, elevation, living conditions, and importantly, diet, are drivers of variation in microbial composition and relative abundance (Muegge et al. 2011; Huus et al. 2021). For example, diet introduces new microbial colonizers into the gut, influencing the overall makeup and stability of the gut microbiome (Quiroga‐González et al. 2021). In addition, soil geochemistry (a factor of environmental exposure) is a strong predictor of microbial abundance in primate systems, likely due to soil's own microbiome dynamics and impact on food nutritional content (Grieneisen et al. 2019). Sociality, group membership, and host‐associated life history traits (e.g., age and sex) can also predict microbial similarity between individuals (Tung et al. 2015; Dill‐McFarland et al. 2019; Risely et al. 2021; Callaghan and Jurburg 2023). Because of the many potential drivers of microbial composition, it is necessary to employ a large‐scale community approach to identifying relationships between individual traits and microbial taxonomic features.

We studied a suite of factors potentially influencing the gut microbiome in a population of wild, free‐living yellow‐bellied marmots (*Marmota flaviventer*) in the Rocky Mountains. This population of marmots is ideal for studying gut microbial variation due to their spatial variability and an available half‐decade dataset consisting of microbial samples from well‐studied individuals (Pfau et al. 2023). This marmot population is subdivided into to higher‐ and lower‐elevation colonies, which differ in the length of the growing season and environmental harshness (Maldonado‐Chaparro, Hubbard, and Blumstein 2015; Philson et al. 2024), with documented differences in life history traits between the colonies. Moreover, prior work found significant associations between individual fitness and social behavior, environmental harshness, and age (e.g., Jamieson and Armitage 1987; Montero et al. 2020; Wey and Blumstein 2012; Maldonado‐Chaparro, Hubbard, and Blumstein 2015; Philson and Blumstein 2023), giving us context to ask about a diversity of variables that might influence the microbiome in this system. Importantly, initial studies of these marmots' gut microbiomes show that variation in microbial abundance is associated with individual mass gain rate, which is necessary for hibernation survival (Degregori et al. 2024), and with social behavior, including associations between alpha diversity and pathogenetic microbes and social isolation (Pfau et al. 2023). Putative consequential associations of gut microbial diversity have been explored in this population, with Pfau et al. (2023) examining the association of microbial diversity and composition with social behavior while Degregori et al. (2024) explored how mass gain rate is related to the abundance of core phyla in marmot microbiomes. However, natural variation in gut microbial is understudied and the role of host‐associated factors as drivers of microbial composition is still unclear, thus further study is required to provide insights into the microbiome‐fitness relationship in this wild system.

In other systems, sex‐based differences in the microbiome are associated with locomotion, foraging, and diet (Xu and Zhang 2021; Wang et al. 2020). For example, microbial composition based on diet is sex‐specific in a wild stickleback population (Bolnick et al. 2014), and sex differences in endocrine systems have been shown to influence microbial composition (Org et al. 2016). As such, we predict an association between marmot sex and gut microbiome composition. Females may have higher abundances of microbes associated with offspring‐rearing, before being able to invest in mass gain, while males may be able to allocate more energy to fat storage (a critical requirement for over‐hibernation survival) earlier in the year than females can. We also predict a relatively small effect size because males and females live in similar environments, share burrows, have similar diets, and are likely are exposed to each other's gut microbes via fecal matter that collectively may homogenize their microbiomes (Videvall, Bensch, and Engelbrecht 2023).

Previous studies show that environmental differences and position on an elevation gradient is associated with microbial abundance (Herder et al. 2021). Consequently, individuals at different elevations change metabolite production and digestive health (Zhang et al. 2023). Therefore, we predict that individuals living in our higher elevation sites (that experience more harsh weather conditions comparatively) will have more variable microbiomes than those at lower elevations.

Social behavior also influences the gut microbiome (Koch and Schmid‐Hempel 2011; Raulo et al. 2021; Sarkar et al. 2020). In our population of marmots, sociality has important fitness consequences and is driven in part by the relative abundance of pathogenic microbes, with *Streptococcus* being negatively related with social connectivity (Pfau et al. 2023). In addition, previous studies have illuminated the significance of social transmission of microbiota, with bacterial transmission through social channels increasing diversity and immune stability over time (Montiel‐Castro et al. 2013; Browne et al. 2017). Due to the relationship between the microbiome and sociality, we predict that the relative abundance of pathogenic microbes will in turn be associated with sociality (quantified with the social network measures degree, strength, closeness, eigenvector centrality, clustering coefficient, and embeddedness) because of transmission that may occur between socially interacting individuals.

Finally, individual age can be associated with the gut microbiome, with older individuals having lower abundances of microbes associated with immune function in rhesus macaques (*Macaca mulatta*; Pallikkuth et al. 2021). Further, gut dysbiosis, or the alteration and imbalance of microbes in the gut, is a known hallmark of aging which can contribute to geriatric degenerative diseases (Lakshminarayanan et al. 2014; Mossad et al. 2021). Thus, microbial dynamics are sensitive to age, further indicating the importance of understanding how age impacts microbial composition (Duan et al. 2019). Given that age can mediate microbial community stability because microbe colonization and growth can improve the resilience and robustness of the microbiome with age (Badal et al. 2020), we predict that age should have a moderate relationship with microbial abundance, with younger individuals having a higher abundance of microbes associated with cultivation and immune system maturation.

To study the factors associated with microbial abundance, we used a structured exploratory approach implemented using *Maaslin2* (Mallick et al. 2021), and controlling for multiple comparisons, that helped us identify host‐associated correlates with a suite of common microbes (Fontaine, Mineo, and Kohl 2022). We explored the role of sex, age, social behavior, and location given their hypothesized and documented relationship with microbial abundance, diversity, and fitness factors in other animal systems (sex: Xu and Zhang 2021; Borgo et al. 2018; age: Moon et al. 2018; Xu and Zhang 2021; environmental conditions: Nguyen, Lara‐Gutiérrez, and Stocker 2021; Sociality: Raulo et al. 2021). As a supplementary analysis, we also used *PiCRUSt* (Douglas et al. 2020) to identify each microbe's functional group to facilitate biological interpretation. By understanding how these intrinsic and extrinsic factors individually influence taxonomic abundance, we can further identify potential drivers of natural microbial variation in the wild.

## 2 Materials and Methods

### 2.1 Site Specifics and Data Collection

We studied yellow‐bellied marmots from 2015 to 2020 (from mid‐April to mid‐September annually) at and around the Rocky Mountain Biological Laboratory in the East River Valley in Gothic, Colorado, USA (38°57′ N, 106°59′ W; ca. 2900 m elevation). Marmots were individually marked and studied at the same colonies annually. Colonies were grouped into two core areas designated as “higher elevation” or “lower elevation” sites (Philson and Blumstein 2023). The higher elevation area experiences harsher weather conditions than the lower elevation areas (Blumstein 2006; Maldonado‐Chaparro, Hubbard, and Blumstein 2015; Van Vuren and Armitage 1991). To uniquely mark individuals and collect microbiome samples, from late‐May (when a majority of the snow has melted, and the marmots have had time to start eating natural vegetation) to mid‐September, we placed Tomahawk live traps at burrow entrances to live capture marmots with the goal of once every 2 weeks. Trapped individuals would be transferred into a cloth handling bag to quantify morphological features, such as body mass, sex, and reproductive status. Each individual receives a unique metal ear tag (Monel self‐piercing fish tags #3, National Band and Tag, Newport, KY) and dorsal fur mark with non‐toxic Nyanzol‐D dye (Greenville Colorants, Jersey City, NJ) to facilitate identification from afar. Since most individuals are born in our study population and we trap nearly all the pups (missing a few that were predated before we could trap them), exact age is known for most individuals. Given most dispersal occurs as yearlings, we can assume most individuals that disperse into our study population are yearlings. However, given some disperses are older, we do not include these individuals in our analysis. The mean lifespan for individuals surviving to adulthood in our population is 3.51 (mean lifespan for all individuals is 1.22 due to most individuals dying within their first year). The marmot study population ranges from pups (age 0) to adults with the longest recorded lifespan being 16 years for a female and 11 for a male (Armitage 2014; Kroeger, Blumstein, and Martin 2021). For our analysis, pups were excluded because they emerge halfway through the year and thus lack whole season data. Our study contained yearlings (aged 1) and adults (age 2–10). The age distribution was skewed younger, with individuals aged 2 and 3 comprising 75% of our dataset and individuals aged from 4 to 10 comprising the remaining 25% of our dataset. To obtain microbiome samples, we collected fecal samples from the traps or during handling, which were then placed into a plastic resealable bag and immediately put on ice before being transferred to a −20°C freezer to await processing.

We conducted behavioral observations using binoculars and spotting scopes from 20 to 150 m away, distances that limited observer effects on subjects while maximizing the ability to quantify social behavior (Blumstein, Wey, and Karisa 2009). We conducted observations during times of peak marmot activity (0700–1000 and 1600–1900 h; Armitage 1962), recording and classifying all social interactions as either affiliative (e.g., play, allogrooming) or agonistic (e.g., fighting, chasing). In addition, we recorded the specific individuals initiating and receiving each interaction, as well as the date, time, and location of each interaction. We identified social groups based on space‐use overlap and a community detection algorithm, MapEquation (Rosvall and Bergstrom 2008; Rosvall, Axelsson, and Bergstrom 2009), and calculated social network measures from weighted and directed affiliative social interactions between known yearlings and adults. We calculated degree, strength, and closeness to quantify individual connectivity, and we calculated eigenvector centrality, clustering coefficient, and embeddedness to quantify individual position within their group (see Pfau et al. 2023 for full detail on our social network methods). We studied marmots under the research protocol ARC 2001–191‐01 (approved by the Animal Care Committee at UCLA on 13‐May‐2002 and renewed annually), under approved protocols through the Rocky Mountain Biological Laboratory, and permits issued annually by Colorado Department of Parks and Wildlife (TR‐917).

### 2.2 Microbiome Data Processing

Microbiome data collection and analysis followed Degregori et al. (2024). Briefly, we isolated bacterial DNA from 286 fecal samples collected from 143 unique individuals using the Qiagen Powersoil Extraction Kit following manufacturer protocols. We generated 16S DNA libraries by amplifying the V4 region of the 16S rRNA gene (Caporaso et al. 2011) DNA by PCR using Qiagen Multiplex PCR kits, using primers 806R (5′‐GGACTACHVHHHTWTCTAAT) and 515F (5′‐GTGCCAGCMGCCGCGGTAA). Following indexing, Laragen (Culver City, California, USA) pooled and quantified our samples to create libraries with equimolar sample concentrations. Multiplexed libraries were paired‐end sequenced (300 bp per sequence) on an Ilumina Miseq v3 at Laragen Sequencing yielded a total of 20,839,221 raw sequencing reads. Overall, original sample sequencing depth ranged from four reads to 235,203 reads. To account for both field and lab contamination, we sequenced an extraction blank and a PCR blank which both returned fewer than 10 reads each.

We analyzed the resulting sequences using QIIME2 (version 2019.9; Bolyen et al. 2019). First, we imported raw forward and reverse reads and visualized the demultiplexed sequences to determine ideal cutoffs for truncation (Bolyen et al. 2019). We then conducted quality control using the QIIME2 DADA2 denoising tool and rarefied data to a minimum depth of 1000 reads, which yielded a final set of 4,529,579 reads across 286 samples from 143 unique individuals. Finally, we summarized the denoised data in an Amplicon sequence variance (ASV) table and determined microbial community diversity indices using QIIME2 diversity tools.

We used *phyloseq* (version 1.38.0; McMurdie and Holmes 2013) package in R (version 4.1.3; R Development Core Team 2023), to further filter and clean the data. Specifically, we removed reads belonging to the phyla of *Eukaryota* and *Cyanobacteria* as well as erroneous hits for Mitochondria and Chloroplast. This was done to ensure that only microbes from marmot hosts were included in the data, not microbes that likely originated from digested plant materials in the fecal samples (Ando et al. 2018). Any unassigned phyla were also filtered out of the dataset. For each taxonomic level, the counts across all samples were taken to obtain relative abundance metrics for all runs. Any duplicate feature IDs were also removed, which yielded an observation measure of 236 samples. These values were then used to calculate a weighted average of all microbe abundances in the marmot gut microbiome.

### 2.3 Data Analysis

PERMANOVA tests were performed to test for the overall changes in community composition based on host‐related factors, such as sex, age, valley location, and six measures of social network measures (degree, strength, closeness, eigenvector centrality, clustering coefficient, and embeddedness) (Table 1). A combined PERMANOVA test was performed for each of these factors against total bacterial community composition. After checking collinearity using variance inflation factors in R package “car”, clustering coefficient and closeness were found to exhibit high collinearity and thus clustering coefficient was tested separately (Fox and Weisberg 2019). Thus, two tests were performed using 999 permutations and the bray method in the R package “vegan” (Oksanen et al. 2024). We used *MaAsLin2* (Microbiome Multivariable Associations with Linear Models; Mallick et al. 2021) to fit generalized linear models to analyze relationships between each assigned relative abundance with our host‐associated factors (see Dallas et al. 2024 for a similar approach). *MaAsLin2* is a tool for determining multivariable associations between identified fixed effects and all taxonomic levels, while also accounting for the multiple comparisons that come with exploring the broad diversity of the microbiome. Fixed effects included age, valley position (higher elevation or lower elevation), sex, social network measures (degree, strength, closeness, eigenvector centrality, clustering coefficient, and embeddedness), and social group size (see Supplemental Materials; Table 1 for a breakdown of this sample size based on fixed effects). To further account for intraindividual and interannual variation, we included individual marmot ID and year as random effects. We log‐transformed and standardized (mean‐centered and divided by one SD) group size and performed centered log ratios (CLR) to the compositional microbiome data (Gloor et al. 2017; Quinn et al. 2019) to better meet model assumptions. We determined statistical significance with the corrected q‐value, which is a conservative statistical metric that accounts for a genomewide false discovery rate (Storey and Tibshirani 2003). Residuals and VIF were checked to ensure Gaussian model assumptions were met.

**TABLE 1:** Beta diversity results from a PERMANOVA for each measure of sociality and individual attributes (i.e., sex, age class, and location).

Measure	df	Sums of Sq	*F*‐Model	*R* ^2^	*p*
Age	1	0.962	2.0626	0.008	0.001
Sex	1	0.546	1.1708	0.00454	0.007
Valley location	1	0.588	1.2597	0.00489	0.002
Degree	1	0.535	1.1458	0.00445	0.012
Strength	1	0.491	1.0519	0.00408	0.135
Closeness	1	0.463	0.9916	0.00385	0.51
Eigenvector centrality	1	0.465	0.9961	0.00387	0.443
Clustering coefficient	1	0.492	1.049	0.00410	0.131
Embeddedness	1	0.471	1.011	0.00391	0.356

To group relevant microbes by function, we used *PiCRUSt* (Douglas et al. 2020) to identify the functions of 16s amplicon sequences. Functional potential was determined using ASV values, allowing for direct comparison with 16S gene sequences from reference genomes. We annotated descriptions of functional pathways using Metacyc pathway functions (EC accessions; Kanehisa and Goto 2000) and aligned feature IDs in the stratified *PiCRUSt* with taxonomic naming assignments to identify likely functions for each microbe type. Amplicon sequence variants were subset based on significant microbes identified using the *MaAslin2* program (Phyla: [*Tenericutes, Firmicutes, Proteobacteria*], Class: [*Mollicutes*], Family: [*Lachnospiraceae, Rikenellaceae*], and Genus: [*Ruminococcus*]). To reduce reads for subsequent analysis, we used the functional relative abundance scores to identify the top ten most abundant enzymatic functions for each microbe and identified primary functional groups to frame the interpretation and discussion of results from the *MaAslin2* model results.

### 2.4 Post Hoc Analysis

To further understand the potential factors influencing the significant microbes identified from the *MaAslin2* analysis, we fitted two post hoc linear mixed effect models in *lme4*. First, centered log ratios (CLR) were performed to remove constraints on the microbe compositional data (Gloor et al. 2017; Quinn et al. 2019). Then, we fit eight models to determine the relationship between seasonality (day of the year) and relative abundance of the ten microbes determined to be significant from the *MaAslin2* analysis. These models included fixed effects (day of the year, age class, valley position, and sex) and random effects (year, individual ID) to account for additional variation. Lastly, to understand if birth and pup rearing partly explained the sex differences in microbial variation, we fitted eight models to explore if female lactation (a binary *yes* or *no* value) is related to the relative abundance of all microbes determined to be significant from the *MaAslin2* analysis. Out of 138 females in our dataset, 51 females were lactating. Fixed effects included lactation status, age class, valley position, and July body mass, and random effects included year and individual ID.

## 3 Results

### 3.1 ASV Clustering and Species Taxonomy Analysis

**FIGURE 1:**
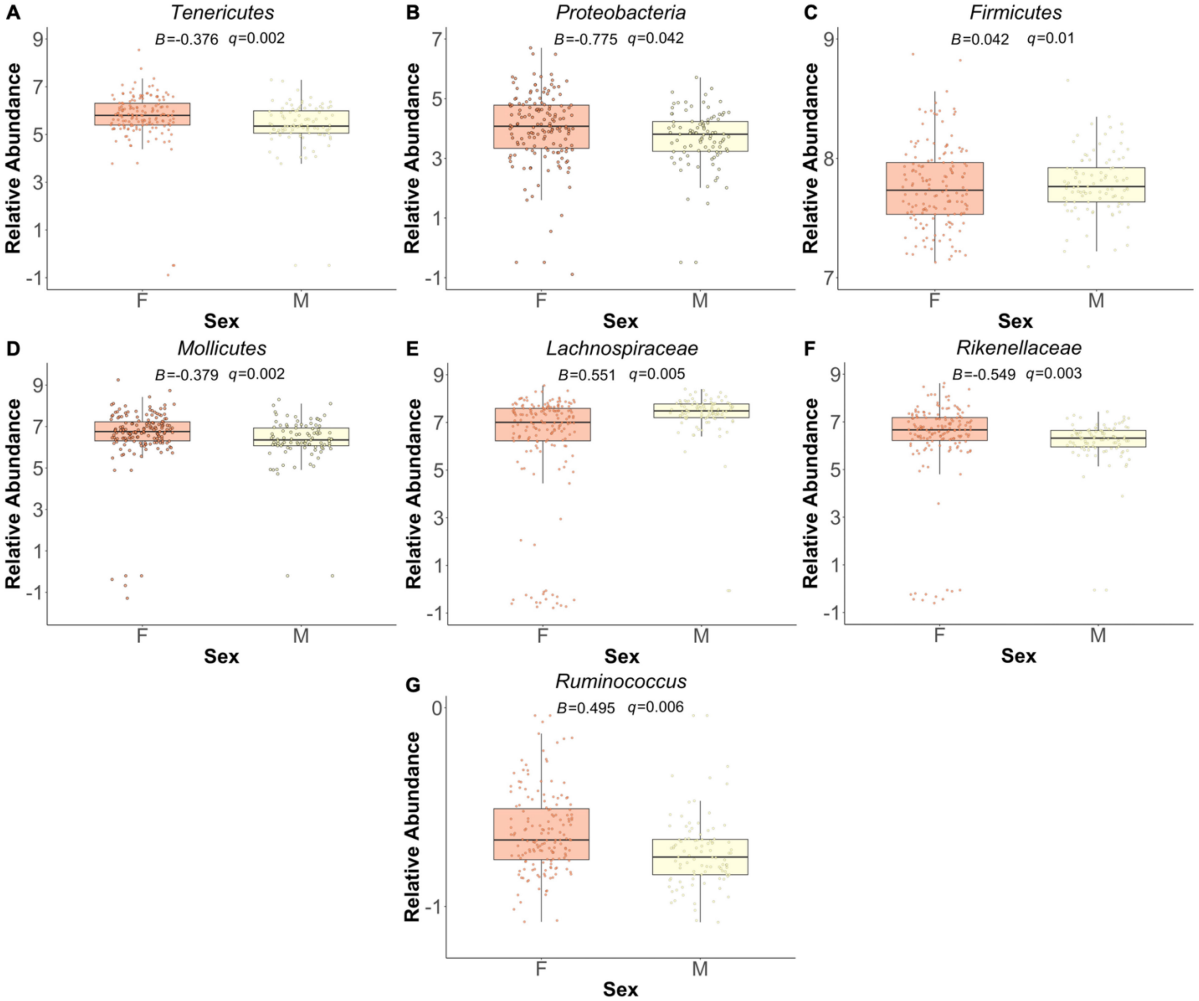
Statistically significant relationships between sex and the relative abundance of microbes across all taxonomic levels. (A) phylum Tenericutes; (B) phylum Proteobacteria; (C) phylum Firmicutes; (D) class Mollicutes; (E) family *Lachnospiraceae*; (F) family *Rikenellaceae*; and (G) genus *Ruminococcus*. These relationships were identified from linear mixed models and thus boxplots here do not represent the marginal effects of this relationship.

After filtering for quality assurance, the final dataset yielded 4,529,579 reads across 286 samples from 143 unique individuals (with 78 being sampled in two or more years). The dataset used here was identical to the dataset used in Pfau et al. (2023), and thereby, the resulting taxonomic analysis mirrors those results. Overall, the marmot microbiome showed high levels of homogenization, with two phyla dominating relative abundance (*Firmicutes* and *Bacteriodetes*, 63.4% [SE = 0.66] and 26.3% [SE = 0.58]). At the class level, *Clostridiales* and *Bacteroidales* (58.3% [SE = 0.11] and 22.5% [SE = 0.13]) accounted for about 80% of all gut microbes. However, family relative abundances were more variable, with *Ruminococcaceae* having the highest relative abundance (28.8%, SE = 0.25), followed by *Muribaculaceae* (14.1%, SE = 0.01), *Lachnospiraceae* (6.2%, SE = 0.31), *Bacteroidaceae* (3.2%, SE = 0.081), and *Rikenellaceae* (2.2%, SE = 0.11). Due to decreasing taxonomic resolution at lower levels, genus and species taxonomic assignments observed higher rates of unassigned taxa. After filtering out unassigned values we observed at the genus level, that *Oscillospira* spp. accounted for 13.8% of all assigned microbes (SE = 1.17), *Bacteroides spp*. made up 13.5% (SE = 0.081), *Ruminococcus* spp. accounted for 12.2% (SE = 0.073), and *Coprococcus* spp. (SE = 1.23). *Akkermansia* spp. (SE = 0.081). *Parabacteroides* spp. (SE = 0.13). *Anaeroplasma* spp. (SE = 0.257) and *Clostridium* spp. (SE = 0.040) together accounted for the other 23.2% of high abundance microbes assigned (Figure 1E). At the species level, *Ruminococcus bromii* was the species in highest abundance at 21.1*%* (SE = 0.29), *Ruminococcus flavefaciens* and *Clostridium colinum* also had high abundance relative to other assigned microbes, with 19.5% (SE = 0.083) and 11.8% (SE = 0.14), respectively The skew of microbial distribution toward a select few microbes across different levels and across multiple individuals suggests low overall diversity of gut microbiota and high homogenization in this wild population of marmots. The overall bacterial analysis using PERMANOVA tests yielded nine total variables, of which four exhibited significant differences in abundance across all bacterial taxa (Table 1).

### 3.2 Relative Microbial Abundance Varies Across Host‐Associated Factors

**FIGURE 2:**
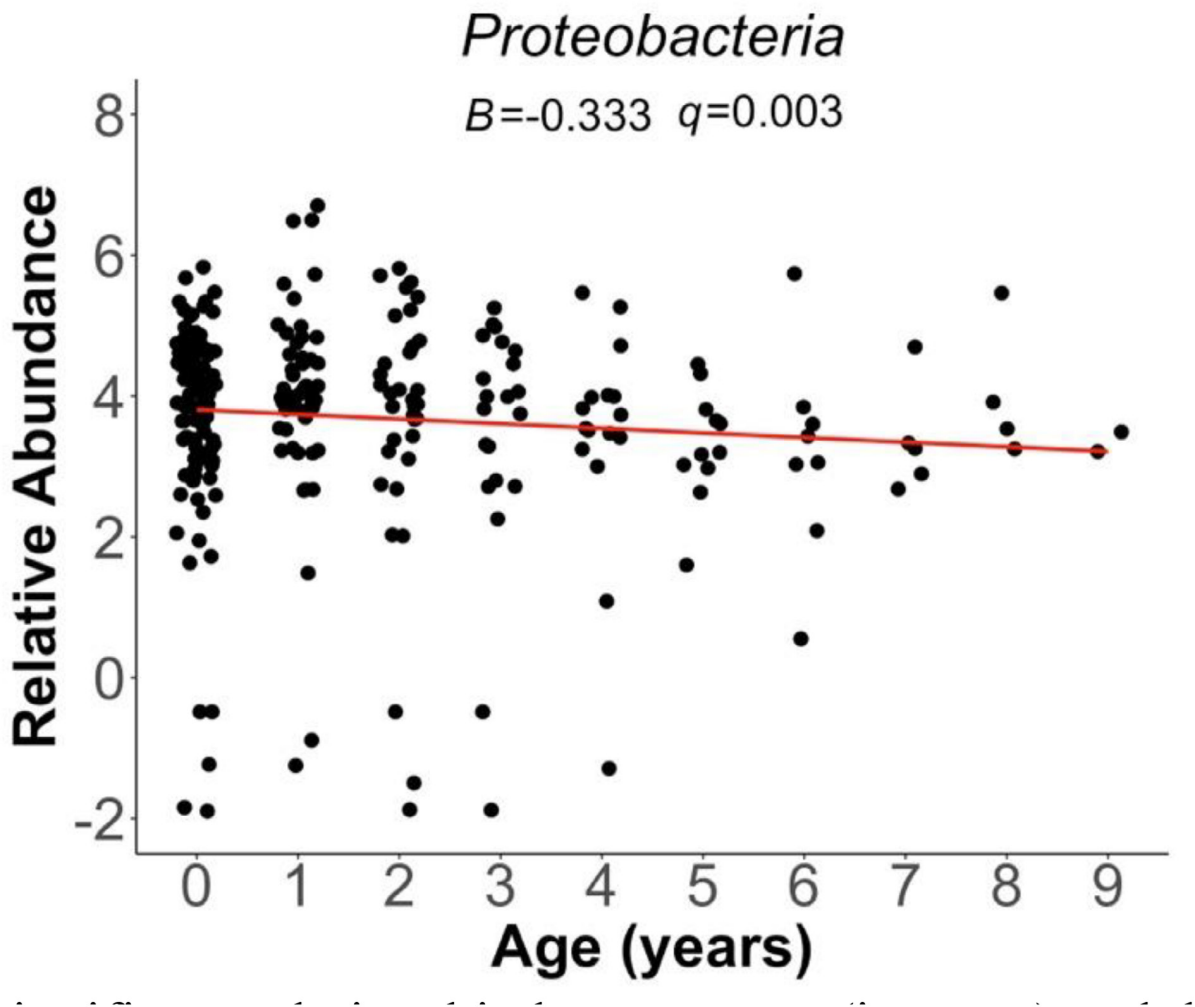
Statistically significant relationship between age (in years) and the relative abundance of the phylum *Proteobacteria*. Red line above showcases the best fit. Age zero represents newly born pups that have weaned from the burrow.

Among the 911 microbes identified across all taxonomic levels, a large majority did not exhibit a statistically significant relationship to host‐associated factors. Specifically, we did not observe any significant relationships between valley location and the measures of sociality with bacterial abundance. Results from the *MaAsLin2* analysis indicate sex‐specific variation in microbial relative abundances. At the phyla level, males had a negative association with *Tenericutes* (*B* = −0.376, *q*‐value = 0.002; Figure 1A) and *Proteobacteria* (*B* = −0.775, *q*‐value = 0.042; Figure 1B). *Firmicutes* relative abundance was positively associated with males (*B* = 0.042, *q*‐value = 0.01; Figure 1C). At the class level, *Mollicutes* class exhibited lower abundance in males and higher abundance in females (*B* = −0.379, *q*‐value = 0.002). At the family level, *Lachnospiraceae* (*B* = 0.551, *q*‐value = 0.005; Figure 1D) abundance was positively associated with males. However, *Rikenellaceae* (*B* = −0.549, *q*‐value = 0.003; Figure 1E) relative abundance was modestly associated with sex, exhibiting higher abundance in males than females. The relative abundance of the genus *Ruminococcus* was positively associated with males (*B* = 0.495, *q*‐value = 0.006; Figure 1F). Lastly, at the phyla level, only *Proteobacteria* was associated with age, exhibiting a negative relationship (*B* = −0.333, *q*‐value = 0.003; Figure 2).

### 3.3 Functional Pathways of Microbial Abundance Explain Variation in Host‐Associated Factors

By analyzing the MetaCyc abundance pathways, functional differences were observed between taxa across all levels. Microbes were subset to reflect the important taxa, as determined by *MaAslin2*. Across all taxonomic levels, enzymes associated with catalyzed reactions needed for epigenetic variance and cell–cell signaling were present and in high functional abundance across all taxa. However, metabolic variation differed across taxonomic levels. Phylum *Tenericutes*, class *Mollicutes*, and family *Rikenellaceae* contained high functional abundances of enzymes associated with weight loss, the production of short chain fatty acids (SCFAs), and synthesis of key molecules needed for accelerated metabolism (pyruvate synthesis) (den Besten et al. 2013). In contrast, *Firmicutes* contained enzymes associated with weight gain (Degregori et al. 2024). Two taxa at the family level, *Lachnospiraceae* and *Ruminococcus*, were associated with enzymes that aid in digestion of complex materials. One phylum, *Proteobacteria*, was also had high functional abundance of enzymes associated with inflammatory regulation (Table S2).

### 3.4 Lactation Explains Variation in Microbe Functional Characteristics, but Seasonality Likely Does Not

**FIGURE 3:**
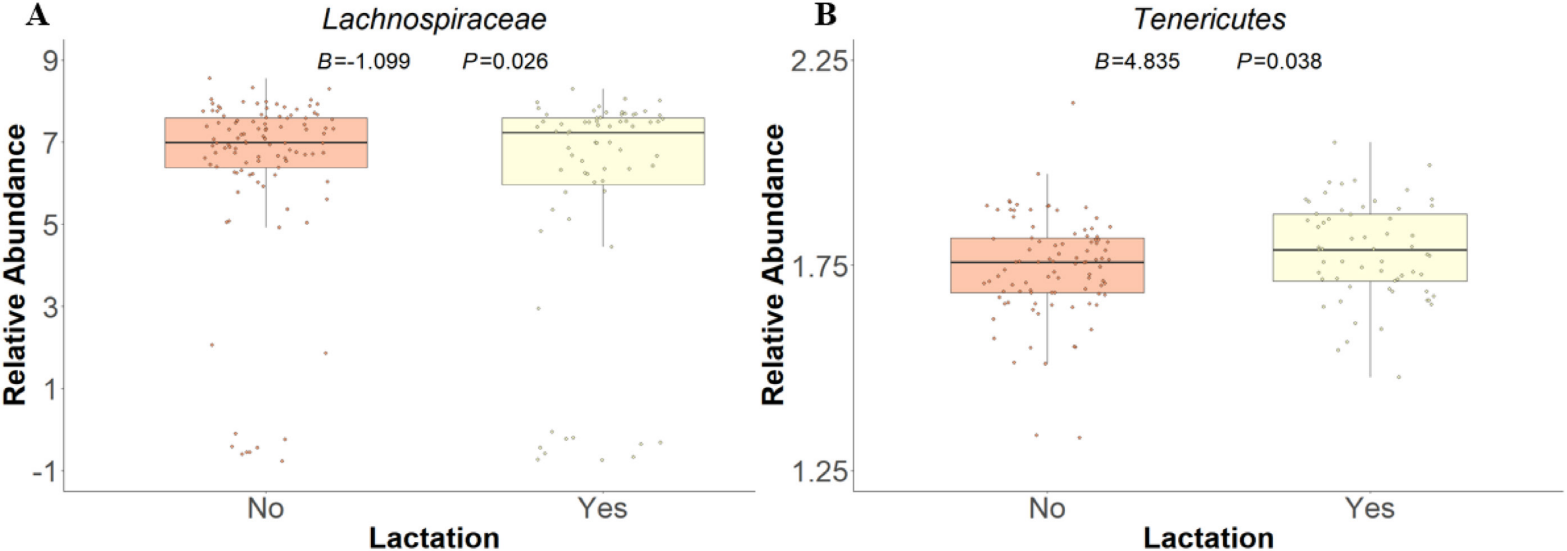
Statistically significant post hoc relationships between lactation status and the abundance of two microbes (family *Lachnospiracea*e (A) and Phylum *Tenericutes* (B)). Lactation represents if females lactated during the year, a strong indicator of reproductive success. These relationships were identified from linear mixed models and thus boxplots here do not represent the marginal effects of this relationship.

From the post hoc analysis within females (72 lactating females out of 193 females in total), lactation was positively associated with *Tenericutes* (*B* = 4.835, *p* = 0.038; Figure 3B), a phylum associated with higher metabolic activity and SCFA production. However, lactation was negatively associated with *Lachnospiraceae* (*B* = −1.099, *p* = 0.026; Figure 3A), a microbial family associated with the metabolism of complex materials. In addition, mass gain was negatively associated with the abundance of *Lachnospiraceae*, which could indicate an inverse relationship between energy‐intensive lactation and over‐summer mass gain. Seasonality was not statistically significant with most microbial abundance in either sex, suggesting that the abundance of most microbial taxa likely does not vary across the active season. The genus *Ruminococcus* did have a statistically significant but very week negative relationship (*B* = −0.002, *p* = 0.008), becoming less abundant in females across the year.

## 4 Discussion

Our study using a multi‐year microbial dataset from a wild marmot population revealed significant sex‐ and age‐mediated variation in microbial composition across multiple taxonomic levels. Through measuring host‐associated factors, we observed that males had a higher overall proportion of microbes that are preliminarily associated with functional enzymes responsible for fat retention and digestion of complex materials while females had higher abundances of microbes associated with functional enzymes shown to improved metabolic energy and weight loss. Since functional characteristics are classified to the closest match in the KEGG system, all functional descriptions may be omitting key functions that have not been shown in understudied wild taxa and thus, associations to functional enzymes are preliminary results. While the extent of sex and age‐related factors driving microbiome composition is still emerging, an increasing number of studies have identified the role of host‐associated factors (sex, age, location within a population, and social behavior) in determining the taxonomic composition of gut microbiomes (sex: Xu and Zhang 2021; Valeri and Endres 2021; age: Moon et al. 2018; Xu and Zhang 2021; environmental conditions: Nguyen, Lara‐Gutiérrez, and Stocker 2021; sociality: Raulo et al. 2021). Our results suggest that factors associated with host features, such as age and sex, outweigh factors related to an individual's environment, such as valley location (i.e., environmental harshness) and sociality.

### 4.1 Sociality and Valley Location Do Not Explain Variation in Microbial Abundance

Out of the 991 microbes tested using *MaAsLin2*, and after accounting for multiple comparisons (via the corrected q‐value), approximately 1% of the microbes were significantly associated with some putative drivers. Surprisingly, sociality and valley location (i.e., environmental harshness) explained no variation in microbial abundances, potentially due to similar diets across our study population (Frase and Hoffmann 1980; Edwards 1997; Evans, Williams, and Blumstein 2021) reducing the overall variance in marmot microbiome composition given microbial acquisition and diversity is strongly associated diet (Edwards 1997; Leshem, Segal, and Elinav 2020; Renelies‐Hamilton et al. 2021; Evans, Williams, and Blumstein 2021).

While features of the microbiome (diversity and abundance) were associated as potential drivers of social behavior previously in this system (Pfau et al. 2023), here we did not observe this relationship bidirectionally given sociality had no significant relationship as a potential driver of microbial composition. Taken together, these two results may indicate that the brain‐gut axis, or the relationship between the gut microbiome and neurological processes, may be unilateral in this system, with social behavior having limited effect on microbial composition (but the microbiome influencing social behavior). Our results here do not support our a priori hypothesis, which was supported by previous studies who have exhibited microbial similarity between socially interacting individuals in other rodent systems (Raulo et al. 2021). This finding may be because yellow‐bellied marmots share burrow systems, forage in similar locations, and consume very similar diets, and thus they may ingest each other's feces at higher rates than other rodents (Armitage 2014, 1979). This potential coprophagia may homogenize their gut microbiome (as seen in captive co‐housed rodents; McCafferty et al. 2013), reducing the significance of any potential social transmission of microbes. Further, Raulo et al. (2024) also demonstrated the importance of the category of the microbe (anaerobic vs. aerobic) in determining microbial transmission since they observed only anaerobic microbes were significantly impacted by social behavior. While neither anaerobic nor aerobic microbes were impacted by social behavior in our study, oxygen dependence may drive variance beyond social behavior in our dataset, potentially impacting the overall significance of the associations. In all, our results here suggest that social relationships may be less important for microbial transmission than other factors, a suggestion that could open doors to exploring non‐social mechanisms for pathogen acquisition in this wild population (see also Wey and Blumstein 2012).

Similarly, environmental harshness, as measured by our proxy of valley position, was not associated with microbial variation. These results do not align previous studies that show increased microbial diversity and compositional variance across elevational gradients (Nguyen, Lara‐Gutierrez, and Stocker 2021). However, this result may be due to homogenization of lifestyle across the population. Previous studies demonstrate that diet has a strong influence on gut microbiomes (Muegge et al. 2011; Miyake et al. 2015). Thus, regardless of colony location and elevation, it is possible that similarity in diet regimes overwhelms the influence of environmental conditions, leading to a lack of a relationship between locality and microbial abundance.

### 4.2 Sex and Age‐Mediated Differences in Microbial Abundances across Multiple Taxa

Sex was modestly associated with the relative abundance of multiple microbial taxa. However, the direction of the relationship varied across microbial taxa, indicating that microbial enzymatic function may be associated with marmot sex. Most of the functional characteristics of the microbes with the highest abundance were in some way related to epigenetic stabilization and respiration efficiency, but metabolic‐associated enzymes were also found in high relative abundance.

Sex‐related variation in microbial relative abundance could be due to hormonal differences between males and females as endocrine function can be associated with microbiome‐specific differences between males and females (Org et al. 2016). Further study into the mechanistic impacts of hormone abundance with microbial abundance is required to corroborate this finding in this system (Valeri and Endres 2021). Previous studies have shown the importance of sex in shaping the taxonomic composition of gut microbiomes and suggested an impact of sex hormones on maintaining this sex‐mediated variation throughout the lifespan (Org et al. 2016; Sun et al. 2021; Santos‐Marcos et al. 2023). Differences in gut microbial composition between males and females in our system may therefore reflect the functionality of the microbe or microbial group. For example, microbes associated with the production of short chain fatty acids and metabolite synthesis (*Tenericutes*, *Mollicutes*, and *Rikenellaceae*) were more abundant in females, indicating that females may have higher enzymatic function responsible for food intake regulation and accelerated metabolic activity, potentially due to lactation investment (Sun et al. 2021). Conversely, males had greater abundance of *Firmicutes*, a phylum associated with mass gain in this species, as well as enzymes required for fat retention (Degregori et al. 2024).

The primary explanation behind the sex differences in microbial abundance we show here may stem from the differences in male and female metabolic investment strategies and limitations. While males can invest in mass gain all summer (to maximize the likelihood of surviving the coming winter hibernation; Wey and Blumstein 2012), females may be nursing young, requiring investment into lactation before being able to invest in mass gain for hibernation. Males producing relative less SCFAs (chemical signatures that are associated with reduced inflammation and increased metabolic energy; Sun et al. 2021; den Besten et al. 2013) and producing more microbes associated with mass gain supports this hypothesis. Males also had a higher abundance of *Lachnospiraceae* and *Ruminococcus*, which are associated with enzymes that break down complex materials, such as cellulose, complex sugars, and large protein molecules (La Reau and Suen 2018; Vacca et al. 2020). In contrast, females had a greater relative abundance of microbes from the phylum *Proteobacteri*a. These microbes are associated with enzymes that reduce gut inflammation (Table S2). This relationship may indicate the importance of sex in mediating a healthy microbiome and overall physiological fitness since gut inflammation is energy‐intensive and may increase disease susceptibility (Kirschman and Milligan‐Myhre 2019; Rendina et al. 2021). These relationships further support the idea that female's microbiome are structured for digestibility, nutrient absorption, and metabolic efficiency at the cost of mass gain.

In addition to sex‐mediated differences, age was modestly negatively associated with one phylum, *Proteobacteria*. A previous study in wild voles indicated that juveniles have more *Proteobacteria* (Moon et al. 2018), which is consistent with our finding. *Proteobacteria* is associated with a less stable gut microbiome community and may facilitate the colonization of new microbial taxa (Moon et al. 2018; Rendina et al. 2021). In addition, as host age increases, microbial stability is expected to increase with lower rates of microbial acquisition and colonization (Moon et al. 2018; Rendina et al. 2021). Therefore, younger individuals may have higher abundances of *Proteobacteria* due to the need for a more transient microbiome, where microbes are flushed in and out the gut ecosystem and microbes colonize frequently, whereas older individuals likely have more gut stability and thus reduced *Proteobacteria* abundance.

### 4.3 Post Hoc Determinations of Mechanistic Roles in Taxonomic Composition

To further understand the variation in taxonomic composition across multiple host‐associated factors, we conducted two suites of post hoc analyses. Our first post hoc analysis showed that lactation had a modest association with microbial abundance and mass gain across two taxa (*Lachnospiraceae* and *Tenericurtes*). Whether or not a female produces a litter in a given year can have a large effect in individual behaviors and energy allocation. Females rearing pups typically allocate more energy to lactation than to individual mass gain (Oli and Armitage 2003). Therefore, we expected to see that lactating females would have a higher abundance of microbes associated with fat loss and metabolic energy (i.e., investment in milk) whereas we expected females who did not give birth and need to invest in milk that year to have higher abundance of microbes associated with fat retention. Our findings support these predictions because females who lactated had a higher abundance of *Tenericutes*, a microbe associated with fat loss and metabolism, and a lower abundance of *Lachnospiraceae*, a microbe associated with digesting complex fibers. *Lachnospiraceae* has also been negatively associated with breastfeeding in human studies as well, indicating that the family of microbes does not act as an ideal nutrient for offspring thus further explaining lower levels of this microbe in lactating females (Ma et al. 2020).

Our second post hoc analysis explored the relationship between seasonality and microbial abundance as fat retention demands tend to increase in the later summer months as individuals prepare for hibernation (Ruf et al. 2023). We largely did not see a significant relationship between any microbes and the time within the year, indicating that seasonality (at least during the period we measured it in the middle, not tail ends, of the active season) does not drive microbial composition. This suggests microbes associated with mass gain are important year‐round, understandable given the relatively short (ca 5 months) to gain 3–5 kg of body mass before winter hibernation (Armitage 2014). However, we did observe one very weak negative relationship between day of the year and abundance of the genus *Ruminococcus* in females, a microbe associated with the digestion of cellulose and other difficult to digest materials (Rojas et al. 2021). A relationship opposite than predicted (Ruf et al. 2023), this very week relationship (beta near zero) requires further data deeper into the active season to fully interpret.

## 5 Conclusions

Symbiotic gut microbes are crucial for most species, and understanding the extent and importance of these interactions is necessary to further understand the causes and consequences of fitness in wild animals. While a rapidly growing field, the mechanistic drivers of microbial composition in wild animals remain understudied. Previous literature has identified some of the difficulties of studying free‐living animals and the multiple factors that influence microbial content (Davidson et al. 2020). Due to these challenges, few studies have examined the associations between a large suite of host‐associated factors in hibernating mammals, where metabolic demands are highly seasonal. Building the foundation for understanding these complex associations can aid in developing new experimental designs to test the influence of a broader suite of environmental and host‐associated factors on microbial composition and biological fitness (Sommer et al. 2016). Overall, results show that sex, not social behavior or environmental conditions, have a modest but specific and significant relationship with microbial variation in a wild mammal's gut microbiome. In addition, there was a possible sex‐specific trade‐off for females between offspring investment (via lactation) and individual mass gain (needed for a successful winter hibernation).

Future studies should identify the underlaying mechanisms of these relationships (Raulo et al. 2021). Specifically, the role of endocrine systems may drive fluctuations in microbes over time, a concept that could open doors to developing a comprehensive understanding of what internal mechanisms may contribute to the adaptive changes in microbial abundance. Further, functional pathway analysis using PiCrust2 has multiple limitations that make it difficult to assign bacterial taxa with a specific function. Specifically, the lack of representation of understudied wild species in the KEGG database means there may be missing functional associations from the analysis in addition to performing the analysis at higher taxonomic levels (such as phyla), which is limiting due to the wide array of possible genera and species within a phylum. Thus, further studies from our preliminary bacterial assignments are needed that perform metagenomic sequencing with functional experimentation to fully assess functionality and causality. In determining the internal factors influencing microbial‐endocrine interactions in marmot systems, we can uncover more information about the drivers of marmot health and fitness on a population‐level scale.

Finally, while our study focused on using traditional methods of mixed modeling to examine the gut microbial metacommunity, we cannot understand the scale of community‐level changes without recognizing the microbiome as an ecological system. Future work should also leverage new tools to understand gut microbial communities, such as longitudinal metacommunity analysis (Murillo et al. 2022), to better understand microbe‐host and microbe‐microbe interactions at a finer scale. Incorporating a mechanistic approach to uncovering microbiome‐host relationships as well as population‐level variation in the microbiome can aid in developing a broader picture of the distribution of microbes in wild mammals.

## Author Contributions

**Madison Pfau:** conceptualization (lead), data curation (lead), formal analysis (lead), methodology (lead), project administration (equal), visualization (equal), writing – original draft (lead), writing – review and editing (equal). **Samuel Degregori:** data curation (supporting), methodology (supporting), resources (supporting), writing – review and editing (supporting). **Paul H. Barber:** data curation (supporting), funding acquisition (supporting), resources (supporting), writing – review and editing (supporting). **Daniel T. Blumstein:** data curation (supporting), funding acquisition (lead), resources (lead), supervision (supporting), writing – review and editing (equal). **Conner S. Philson:** data curation (supporting), project administration (equal), supervision (lead), visualization (equal), writing – original draft (supporting), writing – review and editing (equal).

## Conflicts of Interest

The authors declare no conflicts of interest.

## Supporting information


**Table S1.** Summary table describing the age, sex, and valley location distributions across all samples for the marmot dataset.
**Table S2.** The microbes and their top three functional enzymatic pathways (KEGG), as determined by the functional contribution per taxon from *PiCRUSt*. Functional pathways are described with the enzyme name and the relative function derived from mammalian systems. The average functional abundance is provided to describe the relative importance of each microbial function across all taxa.

## Data Availability

Code and data to recreate analysis associated with this project can be found at https://doi.org/10.17605/OSF.IO/V4FGR.
