# Supplementary material for: Differences in Gut Microbes Across Age and Sex Linked to Metabolism and Microbial Stability in a Hibernating Mammal
Source: Ecol Evol. 2024 Nov 10;14(11):e70519. doi: 10.1002/ece3.70519 (PMC11550910; doi:10.1002/ece3.70519)
Supplement: Supplementary file 1 — Table S1. Summary table describing the age, sex, and valley location distributions across all samples for the marmot dataset. Table S2. The microbes and their top three functional enzymatic pathways (KEGG), as determined by the functional contribution per taxon from PiCRUSt. Functional pathways are described with the enzyme name and the relative function derived from mammalian systems. The average functional abundance is provided to describe the relative importance of each microbial function across all taxa. [file ECE3-14-e70519-s001.docx]

**SUPPLMENTAL MATERIALS**

|  | **Female** | **Male** | **Total** |
| --- | --- | --- | --- |
| **Overall** | 138 | 83 | 221 |
| **Yearling** | 49 | 46 | 95 |
| **Adult** | 89 | 37 | 126 |
| **Higher-Elevation** | 80 | 51 | 131 |
| **Lower-Elevation** | 58 | 32 | 90 |

***Table 1.*** Summary table describing the age, sex, and valley location distributions across all samples for the marmot dataset.

| **Name** | **Top Three Functional groups** | **Functional Abundance** | **Taxon Abundance Contribution** |
| --- | --- | --- | --- |
| **Phyla** |  |  |  |
|  | *Histidine Kinase*: senses environmental change, accelerates cell-signaling | 12.3% |  |
| *Tenericutes* | *DNA directed polymerase*: Genomic stability, epigenetic variation | 12.3% | 46.5% |
|  | *Pyruvate synthesis:* higher metabolic activity, SCFA production | 8.5% |  |
|  | *Histidine Kinase*: senses environmental change, accelerates cell-signaling | 15.8% |  |
| *Firmicutes* | *DNA directed polymerase*: Genomic stability, epigenetic variation | 15.8% | 85.5% |
|  | *Cysteine Synthetase*: Response to nutrient variance, weight gain | 8.9% |  |
|  | *Peptidylprolyl isomerase*: Regulates inflammation, accelerates protein folding | 10.56% |  |
| *Proteobacteria* | *Histidine Kinase*: senses environmental change, accelerates cell-signaling | 11.7% | 26.6% |
|  | *DNA directed polymerase*: Genomic stability, epigenetic variation | 11.7% |  |
| **Class** |  |  |  |
|  | *Histidine Kinase*: senses environmental change, accelerates cell-signaling | 12.3% |  |
| *Mollicutes* | *DNA directed polymerase*: Genomic stability, epigenetic variation | 12.3% | 63.3% |
|  | *Pyruvate synthesis:* higher metabolic activity, SCFA production | 8.5% |  |
| **Family** |  |  |  |
|  | *Histidine Kinase*: senses environmental change, accelerates cell-signaling | 11.5% |  |
| *Lachnospiraceae* | *DNA directed polymerase*: Genomic stability, epigenetic variation | 12.7% | 54.5% |
|  | *α and β mannosidase*: Regulation of undigested materials, metabolizes cellulose and complex sugars | 9.0% |  |
|  | *DNA directed polymerase*: Genomic stability, epigenetic variation | 13.2% |  |
| *Rikenellaceae* | *NADH:ubiquinone reductase (H(+)-translocating:* Oxidizing agent in cellular processes, required for ATP synthesis, metabolism | 13.2% | 34.2% |
|  | *Alpha-L-fucosidase*: SCFA production, metabolism | 10.6% |  |
| **Genus** |  |  |  |
|  | *Cellulose*: Digestion of cellulose | 13.1% |  |
| *Ruminococcus* | *DNA directed polymerase*: Genomic stability, epigenetic variation | 14.6% | 42.5% |
|  | *Serine-type D-Ala-D-Ala carboxypeptidase*: Biosynthesis of difficult to digest materials | 11.4% |  |

***Table 2***. The microbes and their top three functional enzymatic pathways (KEGG), as determined by the functional contribution per taxon from *PiCRUSt*. Functional pathways are described with the enzyme name and the relative function derived from mammalian systems. The average functional abundance is provided to describe the relative importance of each microbial function across all taxa.
